# Anatomical Changes during Chestnut (*Castanea mollissima* BL.) Gall Development Stages Induced by the Gall Wasp *Dryocosmus kuriphilus* (Hymenoptera: Cynipidae)

**DOI:** 10.3390/plants13131766

**Published:** 2024-06-26

**Authors:** Cheng Wang, Wu Wang, Shijie Zhang, Yu Chen, Yuqiang Zhao, Cancan Zhu

**Affiliations:** 1 Institute of Botany, Jiangsu Province and Chinese Academy of Sciences, Nanjing 210014, China; 17811100884@163.com (C.W.); 2017204015@njau.edu.cn (W.W.);; 2 Jiangsu Key Laboratory for the Research and Utilization of Plant Resources, Nanjing 210014, China

**Keywords:** chestnut gall wasp, gall development, gall morphological features, gall anatomical structure, ROS

## Abstract

This study delved into the larval development and the morphological and anatomical transformations that occur in the galls of chestnut trees (*Castanea mollissima* BL.) and are induced by the chestnut gall wasp *Dryocosmus kuriphilus* Yasumatsu (GWDK) across various stages: initial, growth, differentiation, maturity, and lignification. Chestnut galls in the five development stages were collected. Gall structural characteristics were observed with an anatomical stereomicroscope, and anatomical changes in galls were analyzed with staining and scanning electron microscope techniques. The chestnut gall wasp laid its eggs on young leaves and buds. Chestnut gall wasp parasitism caused plant tissues to form a gall chamber, with parenchyma, protective, and epidermal layers. The development of the gall structure caused by the infestation of the GWDK gall led to the weakening of the reactive oxygen species (ROS) elimination ability of the host. The accumulation of ROS led to cell wall peroxidation, resulting in structural damage and diminished host resistance, and the parenchyma layer exhibited significant nutrient supply and thickening. The thickness of the protective and epidermal layers varied notably across different growth stages. The oviposition of the chestnut gall wasp induced modifications in the original plant tissues, with gall formation being most favorable in young tissues, correlating with the maturity level of the host plant tissues. Variances in the internal structures of the galls primarily stemmed from nutrient supplementation, while those in the external structure were attributed to defensive characteristics. This research contributes a foundational understanding of gall development induced by the chestnut gall wasp in Chinese chestnut, offering valuable insights into the intricate interplay between insect infestation and plant physiology.

## 1. Introduction

Most cynipids (Cynipidae and Hymenoptera) are not considered economic pests, but a notable exception is the chestnut gall wasp *Dryocosmus kuriphilus* Yasumatsu (Cynipidae, Hymenoptera). The GWDK is a significant pest infesting chestnut trees (Castanea sp.) [1].

Galls are neoformed plant structures, induced by organisms such as insects, nematodes, or microorganisms [2]. Gall inducers, especially insects, are usually specific to their host plant species. The anatomy of gall morphotypes are strongly related both to the species of the gall inducer and the species of its host plant [3]. We still know little about the development of galls. Galls are induced to provide both nutrients and shelter for the developing larvae and may also provide protection against natural enemies [3,4], and the same occurs in galls of the GWDK.

Originally native to China, its invasion into other regions has led to substantial economic losses, particularly in key chestnut-producing areas like Hebei and Jiangsu [1]. Both domestic and imported chestnuts in these areas have been severely impacted, directly affecting the economic output of chestnut-growing regions. This pest operates on an annual cycle, with one generation per year, reproducing through parthenogenesis. Adult wasps lay their eggs within the buds of new shoots between June and July, and the newly hatched larvae overwinter within these buds [5]. As spring arrives, the larvae commence feeding, causing damage that culminates in the formation of galls. The invasion by these wasps and the ensuing gall formation disrupts bud development, diminishes leaf photosynthetic capacity, and ultimately leads to a decline in tree vitality, resulting in significant yield reductions of up to 75%. In severe cases, the impact can extend to leaf and branch deformation, stunted growth, or even plant mortality [6]. Galls induced by the chestnut gall wasp serve as crucial hubs for developing larvae, offering sustenance, protection against predators, and insulation from adverse environmental conditions [3]. The formation and persistence of these galls are intricately linked to both the oviposition behavior of the wasps and the feeding activities of the larvae. Because galls are entirely composed of plant tissues, their development is influenced not only by host plant characteristics but also by various environmental factors. The process of gall development typically follows distinct morphogenetic stages—initiation, growth, and maturity [7]. Building upon this understanding, previous studies, such as that by Wang et al. who studied the morphological development process of galls induced by the wasp *Quadrastichus erythrinae*, have delineated gall formation into stages encompassing initial formation, early and late growth, differentiation, maturity, and eventual cracking [8].

Chestnut galls are covered with spherical structures, which are surrounded by lanceolate leaflets. Larvae are sealed in the gall chamber, which are surrounded by a hard shell [1]. Structurally, the location and number of gall chambers in the galls are influenced by the maternal oviposition, whereas gall initiation and maintenance are influenced by the oviposition and larval feeding. Because galls are formed entirely of plant tissues, gall initiation and growth are influenced by host plant traits and by environmental factors. Normal plant development follows the morphogenetic patterns determined by plant meristems, which are changed by the galling stimuli, whereas the rearrangement of gall tissues begins in meristematic tissues such as protoderm, ground meristem, and procambium, leading to an over-differentiation and/or inhibition of some anatomical structures, and sometimes to cell redifferentiation. The gall epidermis remains a single layer, but the cell expansion patterns are slightly modified, becoming periclinally elongated and non-papillose. This alteration is due to cell hypertrophy and hyperplasia in the ground-system cells, increasing the gall volume [9,10]. Annular or laminar collenchyma occurs in the subepidermal layers adjacent to parenchyma cells. The vascular system comprises small vascular bundles within the parenchyma surrounding the larval chamber, predominantly consisting of open collateral vascular bundles. The innermost layer of the gall typically encircles one or more larval chambers, while sclerenchyma cells form a protective sheath around each chamber. Nutritive tissue is arranged around the larval chamber, enclosed within the sclerenchyma sheath [11,12,13,14].

Despite the importance of understanding the gall formation induced by the chestnut gall wasp, relatively few comprehensive studies have been conducted [15,16,17,18]. While previous research has touched upon gall structure and chemical aspects, the nuanced differences in internal gall structure across developmental stages remain largely unexplored. This study aims to bridge this gap by comparing various stages of gall development and elucidating the associated changes in internal structural characteristics. By doing so, it seeks to provide a theoretical framework for future investigations into the intricate dynamics of gall formation induced by the chestnut gall wasp.

## 2. Results

### 2.1. Observations of Gall Morphological Characteristics

**Figure 1:**
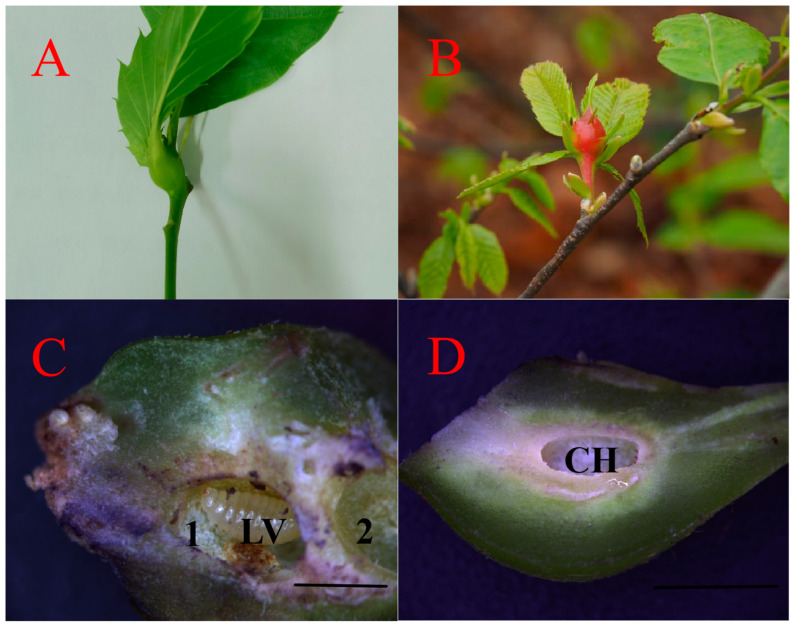
External morphology of chestnut galls induced by GWDK, with longitudinal sections showing gall chambers. (**A**): Early gall of chestnut gall wasp (stage A, initiation); (**B**): Mature gall of chestnut gall wasp (stage D, maturation); (**C**): Multiple gall chambers (Black mark 1, 2) and a gall wasp larva; (**D**): Gall with single chamber. CH: Gall chamber, LV: larva. Scale bars = 500 μm.

Numerous eggs of the chestnut gall wasp (GWDK) were discovered within the tender buds, shoots, leafstalks, and epidermal tissues of chestnut leaves. As larvae fed on those tissues, they induced hyperplasia and deformation, resulting in the formation of nodular galls. Morphological examinations revealed that the galls were covered, spherical structures (Figure 1B), with either multiple closed gall chambers (Figure 1C) or a single chamber hosting multiple larvae (Figure 1D). Galls were green in the initiation stage (stage A), whereas in the maturation stage (stage D), the outer wall of the galls hardened and tended to become lignified, turning red (Figure 1A,B).

### 2.2. Microscopic Observations of Galls

#### 2.2.1. Anatomical Observations with Stereomicroscope

**Figure 2:**
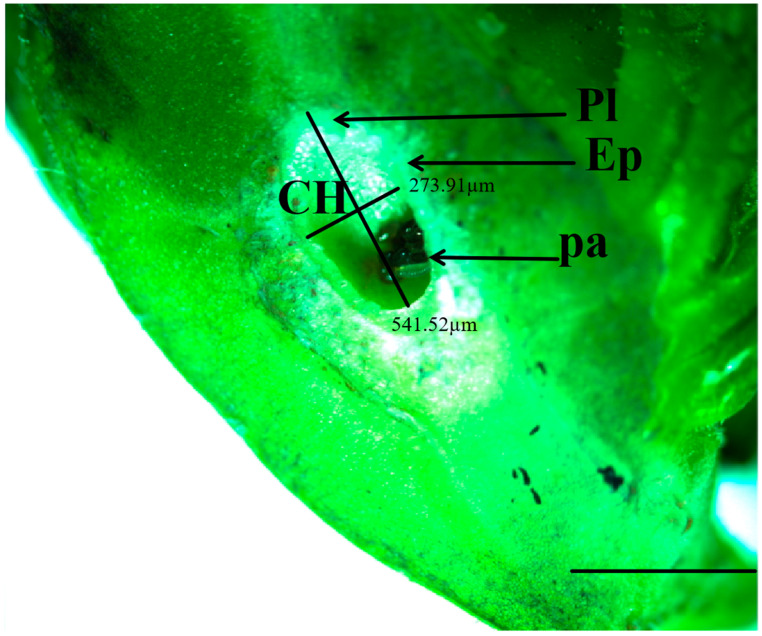
Structures of longitudinal section of stage A chestnut gall induced by GWDK (anatomical stereomicroscope). CH: Gall chamber, pa: Parenchyma layer, Pl: Protective layer, Ep: Epidermal layer. Scale bars = 500 μm.

**Figure 3:**
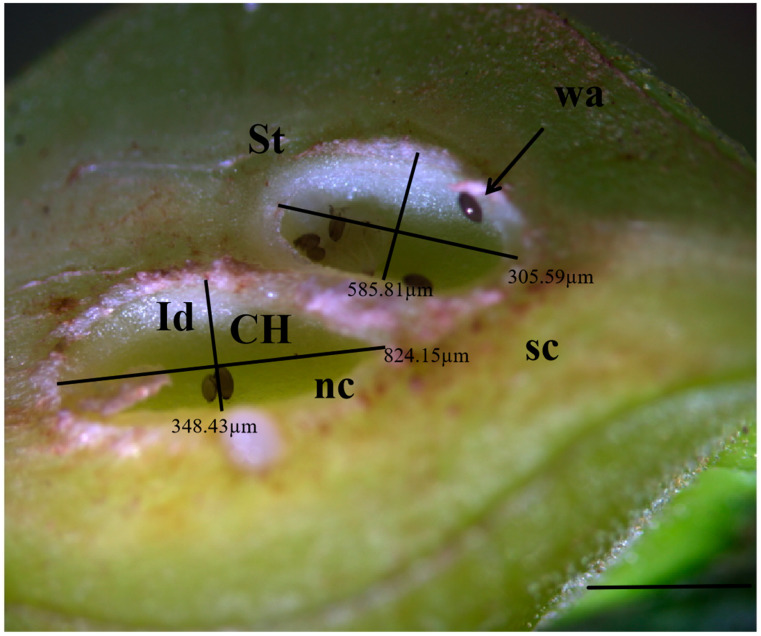
Internal structures of stage A chestnut gall induced by GWDK. CH: Gall chamber, St: Soft tissue with vacuoles, nc: nutritive cells, sc: sclerenchyma epidermal gall layer, Id: internal dense tissue, wa: wasp eggs. Scale bars = 500 μm.

**Figure 4:**
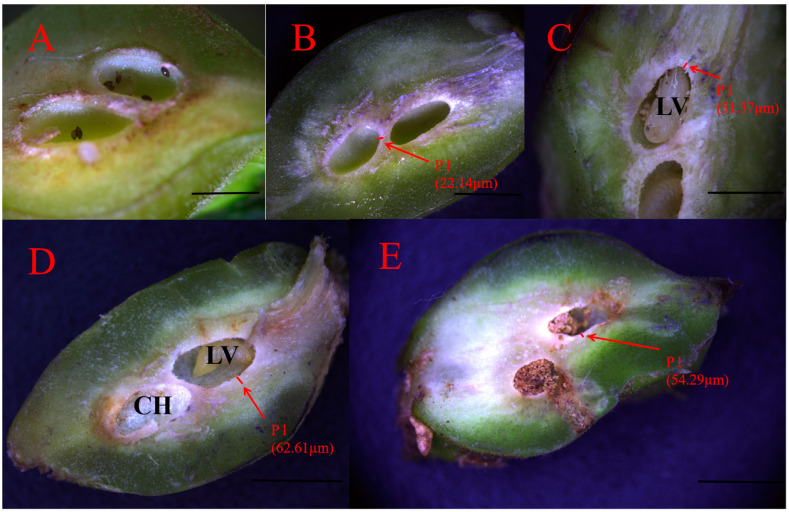
Development of the internal structure of chestnut gall induced by the chestnut gall wasp *Dryocosmus kuriphilus*. (**A**): Stage A (initiation); (**B**): stage B (growth); (**C**): stage C (differentiation); (**D**): stage D (maturation); (**E**): stage E (lignification). The red Pl in the Figure 4B–E represents the protective layer, and the short red line which pointed by the red arrow is the width of protective layer (Pl). CH: Gall chamber, LV: Larva, Pl: Protective layer. Scale bars = 500 μm.

Longitudinal sections of GWDK galls were observed under an anatomical stereomicroscope. Gall structure consisted of four parts: epidermis, protective layer, parenchyma layer, and gall chamber (Figure 2). The epidermal layer is the outermost structure of the gall, which was composed of epidermal cells and a sclerenchyma epidermal gall layer (Figure 3). The makeup of the protective layer, enveloped by the epidermis, was distinctive and primarily consisted of green sclerenchyma cells, soft tissue containing vacuoles, and an inner dense tissue. The layer was light-colored and appeared succulent and gradually expanded during stages B, C, and D (Figure 4B–D). As development progressed to the final stage (stage E), the protective layer transitioned to a darker brown hue (Figure 4E). The parenchyma layer was composed primarily of parenchyma cells, which proliferated significantly during stage B. Concurrently, the inner parenchyma cells disintegrated, forming a nutrient-rich layer adjacent to the outer circumference of the gall chamber (Figure 3). Termed the trophic layer, these cells differed from those of other gall tissue types, with smaller cell space, higher tissue density, less pronounced stratum corneum development, and thinner cell walls. At the same time, the activities of amylase, protease, aminopeptidase, and phospholipase in this layer of cells are higher than those in normal plant cells, and larvae mainly feed on this special layer of cells (Figure 2). The innermost gall chamber was the primary space for GWDK larval movement (Figure 4). The chambers, ranging from 1.0 to 3.2 mm in length and from 1.0 to 2.0 mm in width, contained copious amounts of white-yellow waxen components secreted by GWDK larvae, along with traces of brown-black excreta (Figure 4E).

The entire life cycle of the chestnut gall wasp is completed in the galls, encompassing the egg, larval, and pupal stages. As larvae feed and absorb substantial amounts of nutrients and water, gall tissues undergo a notable transformation, losing chlorophyll and assuming a dark brown hue. By stage D, galls tend to undergo lignification, marking the cessation of larval development because they cease to absorb host plant nutrients. Subsequently, upon exiting the gall chamber and taking flight, multiple exit holes become evident on the outer surface of the galls. Ultimately, the affected twigs with galls wither and die.

**Figure 5:**
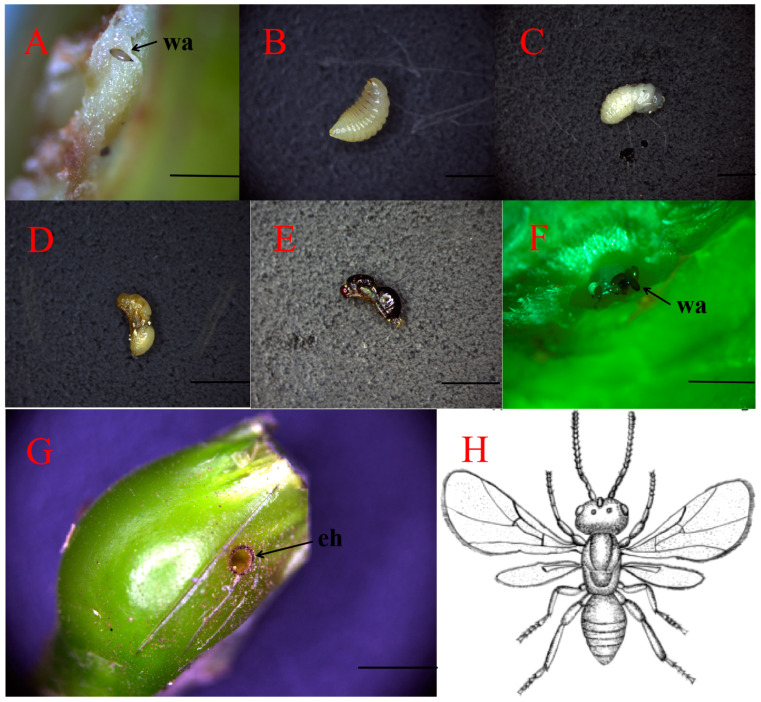
(**A**–**F**): Egg to pupa development of *Dryocosmus kuriphilus*, the chestnut gall wasp (GWDK), and (**G,H**): wasp exit channels on outer and adult stage of GWDK. (**A**–**E**): Developmental stages of GWDK larvae ((**A**): egg; (**B**): larva; (**C**–**E**): pupa; (**F**): GWDK eggs; (**G**): GWDK exit hole on outer gall surface; (**H**): GWDK ink drawing at adult stage. wa: wasp eggs; eh: exit hole. Scale bars = 500 μm.

Larval development within a gall chamber progresses through four discernible stages:

(1) Egg stage: Oval, brown eggs had a smooth surface, with lengths ranging from 0.15 to 0.17 mm and widths from 0.10 to 0.20 mm. Eggs were characterized by a pointed end, slightly enlarged at the apex (Figure 5A,F).

(2) Larval stage: Mature larvae, measuring between 2.5 and 3.25 mm in body length, appeared milky-white, transitioning to yellow-white when nearing maturity. They featured brown mouthparts, distinct thoracic and abdominal segments, and a smooth body texture (Figure 5B).

(3) Pupal stage: Pupae, ranging from relatively round to blunt, measured 2.5 to 3.25 mm in length. In early stages, pupae were milky-white with a slightly yellowish abdomen. As they matured, the pupal bodies turned light brown with a whitish abdomen, ultimately darkening to dark brown immediately before emergence. Brown mouthparts, red compound eyes, and a smooth surface characterized pupae nearing emergence (Figure 5C–E).

(4) Adult stage: Females were glossy and black-brown, with wide heads that were equal in width to that of the thorax. The top of the head was densely covered with small circular patterns between monocular compound eyes and the upper part of the back head. The leading edge of the labial base was curved, whereas the posterior abdomen was smooth, with a nearly oval and raised dorsal surface. The ovipositor tube, observed in an oblique cut of the abdomen, was brown and positioned close to the center of the end of the ventral surface. Adult females had yellowish-brown tarsi and dark brown legs with claws, with hind tarsi being the most developed (Figure 5H).

During gall development, the tissues surrounding the gall chamber rapidly proliferated and expanded, starting from stage A. However, the rapid growth trajectory gradually decelerated when reaching stage C, eventually halting by stage D. By stage E, damage to vascular bundle tissues disrupted water transport, leading to atrophy and desiccation of the gall chambers (Figure 4E). Compared with stage A, the thickness of both the epidermal and protective layers increased significantly by stage B. The parenchyma layer reached its maximum thickness at stage C, surpassing that of stage B by a substantial margin. As the galls developed, the gall chambers expanded, reaching their peak size by stage D.

#### 2.2.2. Paraffin Section Safranin O/Fast Green, Periodic Acid–Schiff, and Reactive Oxygen Species Accumulation Stainings

##### Safranin O/Fast Green and Periodic Acid–Schiff Stainings

**Figure 6:**
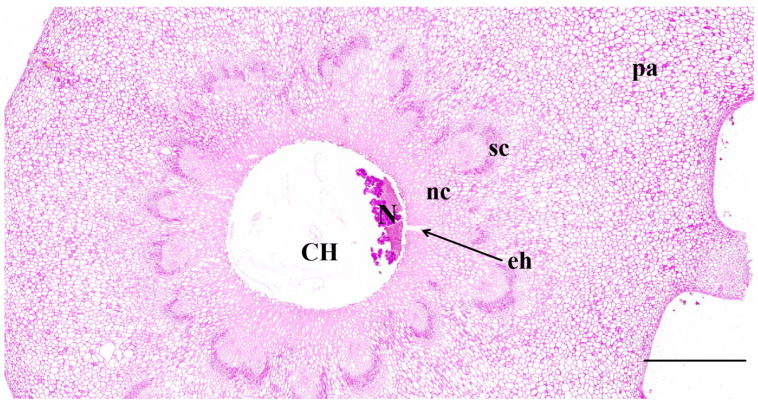
Periodic acid–Schiff staining of longitudinal section of a stage D gall chamber of GWDK. CH: Gall chamber, pa: Parenchyma tissue, sc: Sclerenchyma sheath tissue, nc: Nutrient cells, N: Nutrient layer residue and excrement left by GWDK larvae at exit hole, eh: Preliminary exit hole of GWDK larvae. Scale bars = 500 μm.

The gall chambers were encircled by numerous parenchyma cells (pa in Figure 6), arranged from the outermost layer to the innermost. Beneath the parenchyma layer, sclerenchyma cells formed a distinctive petal-shaped dark ring known as the sclerenchyma sheath (sc in Figure 6). The sclerenchyma cells exhibited hypertrophy, whereas trophic cells (nc in Figure 6) were situated around the chamber and embedded within the sclerenchyma sheath. Comprising large basophilic cells with a loose cytoplasm abundant in euchromatin, the nutritive tissue appeared relatively light in color. Positioned as the innermost cell layer, it enveloped one or more gall chambers (CH in Figure 6).

Stimulated by the oviposition and the feeding activities of GWDK larvae, notable changes occurred in the leaf structure of the host plant, leading to the formation of galls. The galls gradually matured until the departure of adult wasps, a developmental process delineated into five distinct stages.

In stage A, with the introduction of eggs into the host, cells surrounding the medulla were stimulated, resulting in their spread and differentiation into callus-like cells. This transformation was characterized by a reduction in cell size, enlargement of nuclei, increased cytoplasmic density, and a tightly packed arrangement.

**Figure 7:**
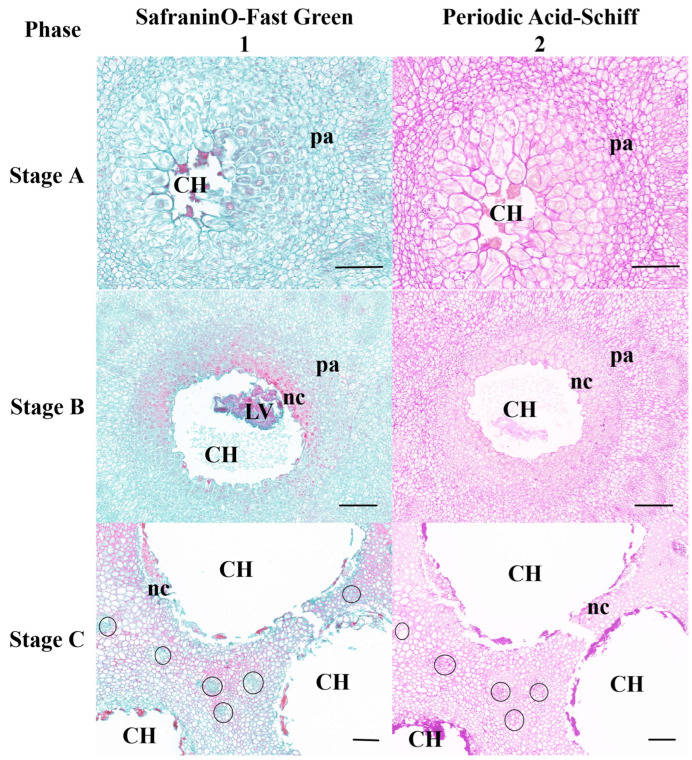

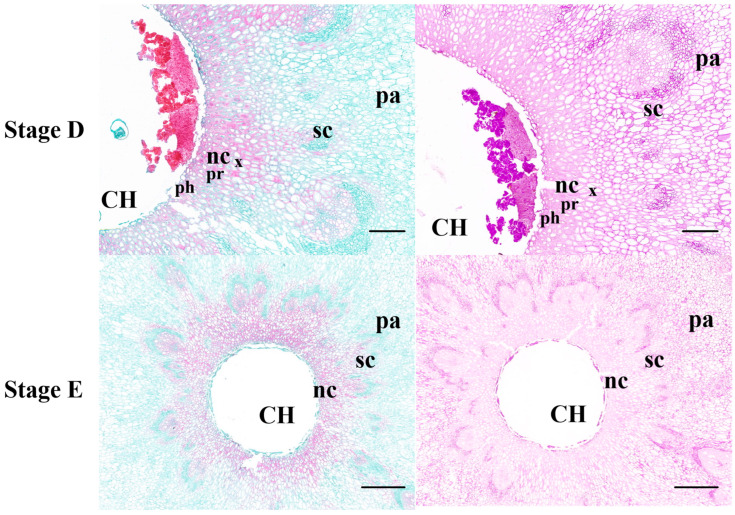
Detection of cellular changes in gall chambers of GWDK during gall development with (1) Safranin O/Fast Green and (2) Periodic acid–Schiff stainings. Stage A (initiation); stage B (growth); stage C (differentiation); stage D (maturation); stage E (lignification). (A1,A2): Parenchyma cells surrounded the initial gall chamber. (B1,B2): Inner parenchyma cells gradually form the nutritive cells. (C1,C2): Each circle indicates a vascular bundle. Note that the larval chamber (CH) is surrounded by scattered vascular bundles. (D1,D2): The procambium in the nutritive cell layer forms phloem inward and xylem outward. (E1,E2): Nutritive cells (nc) lining a larval chamber and adjoining layers of sclerenchyma (sc). CH: Gall chamber, LV: larva, nc: nutritive cells, pa: parenchyma, ph: phloem, pr: procambium, sc: sclereids, x: xylem. Scale bars: A1,A2 = 100 μm; 1/2 (B,C,D) = 200 μm; E1,E2 = 500 μm.

Compression and distortion of the innermost cells led to the formation of one or more small, closed cavities with uneven edges, representing the initial gall chambers (Figure 7(A1)). The deeper purplish-red hue observed in PAS-stained sections indicated a higher carbohydrate content in the corresponding tissues (Figure 7(A2)).

During stages B and C, the parenchyma cells surrounding the gall chamber exhibited an increase in nuclei and cytoplasmic density, resulting in a notable intensification of coloring during stage B (as depicted in Figure 7(B1)). As the larvae of the GWDK became progressively embedded within proliferating parenchyma cells, the inner parenchyma cells dissolved, giving rise to a layer of nutritive cells encircling the gall chamber (as illustrated in Figure 7A,B). Simultaneously, the vascular system began to divide into small vascular bundles within the cortical storage parenchyma surrounding the gall chamber, facilitating nutrient supply from other parts of the host. These tissues served as the primary nutrient source for the larvae and remained undifferentiated (Figure 7(C1,C2)). In instances where the galls coalesced, the larval chambers were separated by several layers of parenchyma cells. However, in cases where the larval chambers were closely situated, vascular bundles were lacking in the fusion area. Throughout stage C, as larval feeding ensued, meristematic activity and cell division increased, accompanied by substantial enlargement of the surrounding cell nuclei. These changes led to a deepening of the purplish-red hue, indicative of increased carbohydrate content, and significant enlargement of the gall chamber (Figure 7(C1,C2)).

In stage D, the growth of gall tissues stopped, GWDK larvae in gall chambers stopped absorbing host nutrients, and the tissues continued to differentiate into nutrient tissues, starch-rich tissue layers, and sclerenchyma cell layers containing lignin (Figure 7(D1,D2)). The parenchyma cells began to lignify, and the inner cells formed a membrane structure. The nutritive cells surrounding the gall chamber continued to grow and accumulate into hardened and cleaved cell tissues, and thus, the inner cells formed phloem, and the outer cells formed xylem, further thickening the outer circumference of the galls. The mature central region of the galls developed into a standard gall chamber with its maximum area and smooth edges (Figure 7(D1,D2)). Amyloplasts and druses were not observed within gall tissues.

In stage E, as the GWDK larvae entered the adult stage, exit holes opened at the top of the galls, penetrating either the upper or lower epidermis to create round exit holes. The cells surrounding the gall chamber underwent lignification, desiccation, and subsequent disintegration, ultimately leading to cell death (as shown in Figure 7(E1)). At this point, the purple coloration faded, and the carbohydrate content decreased (as observed in Figure 7(E2)).

##### Staining for Reactive Oxygen Species Accumulation

Reactive oxygen species (ROS) are pivotal in plant disease development, and understanding their changes in abundance and activity during gall development is crucial for comprehending the mechanisms underlying gall formation and disease resistance breeding. The structure of GWDK-induced galls is linked to the weakening of ROS elimination ability and ROS synthesis. The accumulation of ROS induces cell wall peroxidation, resulting in structural damage and loss of host resistance, thereby promoting gall development.

**Figure 8:**
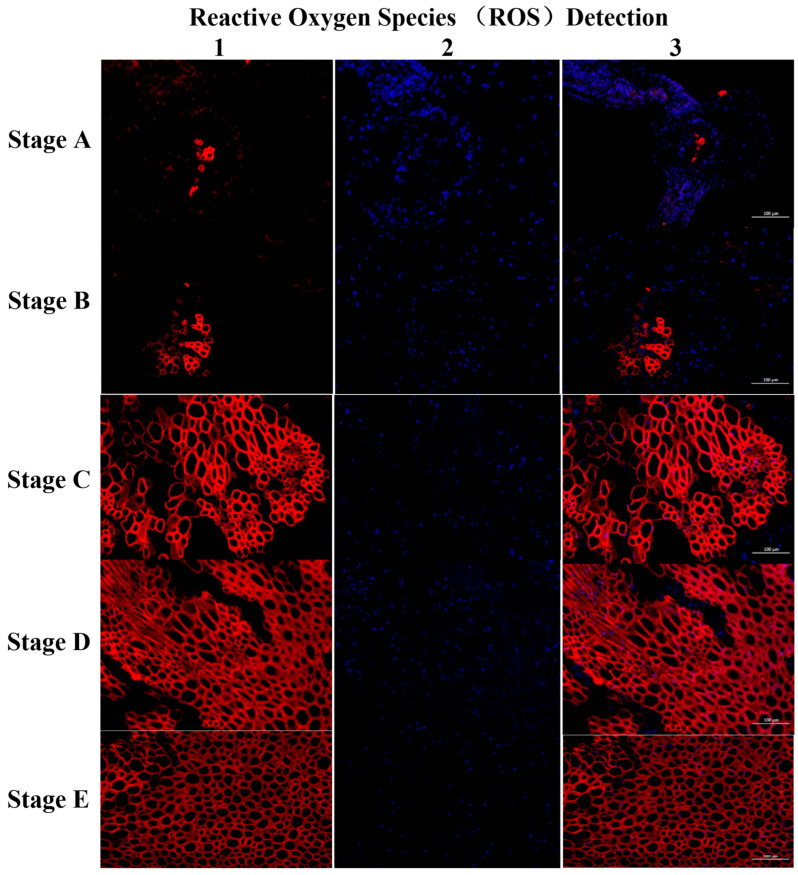
Detection of cellular changes in gall chambers of GWDK during gall development with (1–3) staining for reactive oxygen species accumulation. (Nuclei are depicted in blue in the DAPI channel, while the positive CY3 channel appears red.) Stage A (initiation); stage B (growth); stage C (differentiation); stage D (maturation); stage E (lignification). Scale bars = 100 μm.

In Figure 8, nuclei are depicted in blue in the DAPI channel, while the positive CY3 channel appears red. Reactive oxygen species include reactive oxygen superoxide anions, hydrogen peroxide, and hydroxyl radicals. During stage A, ROS species were scarce (as depicted in Figure 8(A1,A3)), primarily distributed among numerous nuclei (as shown in Figure 8(A2)). Activity began to increase in stage B (depicted in Figure 8(B1,B3)). By stage C, ROS begin to proliferate from the nuclei. ROS accumulation became substantial in the cell wall, exhibiting noticeable regionalization (as indicated by red circles in Figure 8(C1,C3)). In stage D, a significant eruption of ROS occurred, reaching the maximum density, which induced the deterioration of the cell wall (as observed in Figure 8(D1,D3)). In stage E, the intensity of the red color diminished and the ROS content gradually decreased. Concurrently, the cell wall experienced a mass rupture, leading to a sharp decrease in the number of nuclei (as illustrated in Figure 8(E1,E3)).

ROS accumulation can induce host cell wall peroxidation and cell nucleus death, resulting in a gradual loss of host resistance, which promotes gall development.

#### 2.2.3. Scanning Electron Microscopy (SEM)

**Figure 9:**
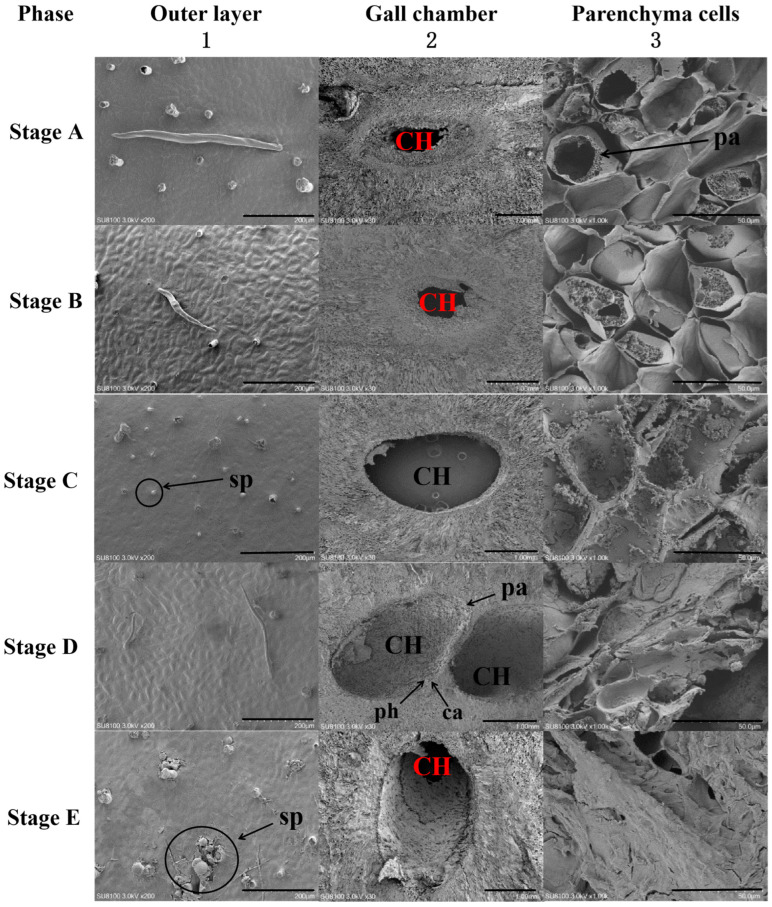
Scanning electron micrographs of the outer epidermis, gall chamber, and parenchyma cells of chestnut galls induced by the chestnut gall wasp *Dryocosmus kuriphilus* at different developmental stages. Stage A (initiation); stage B (growth); stage C (differentiation); stage D (maturation); stage E (lignification). CH: Gall chamber, sp: skin pore, pa: parenchyma, ph: phloem, ca: cambium, Scale bars: 1 (A–E) = 200 μm, 2 (A–E) = 1.00 mm, and 3 (A–E) = 50.0 μm.

In stage A, the SEM results show that the outer upper and lower epidermis layers of the galls exhibited a curling, blistering, and milky appearance with some fine hairs, while the surface remained smooth (Figure 9(A1)).

In stage B, the outer epidermis exhibited roughness with distinct lines and small protrusions, likely due to short-term nutrient and water deficiencies (Figure 9(B1)). During stages C and D, as gall development continued, the nutrient supply function of the gall system seemed to normalize. Consequently, the outer surface became smoother and flatter than in stage B, with fewer protrusions and some damage to skin pores (Figure 9(C1,D1)). By stage E, the outer surface became rougher, displaying outgrowths with proliferative cracking and skin pores ceased to function entirely (Figure 9(E1)).

**Figure 10:**
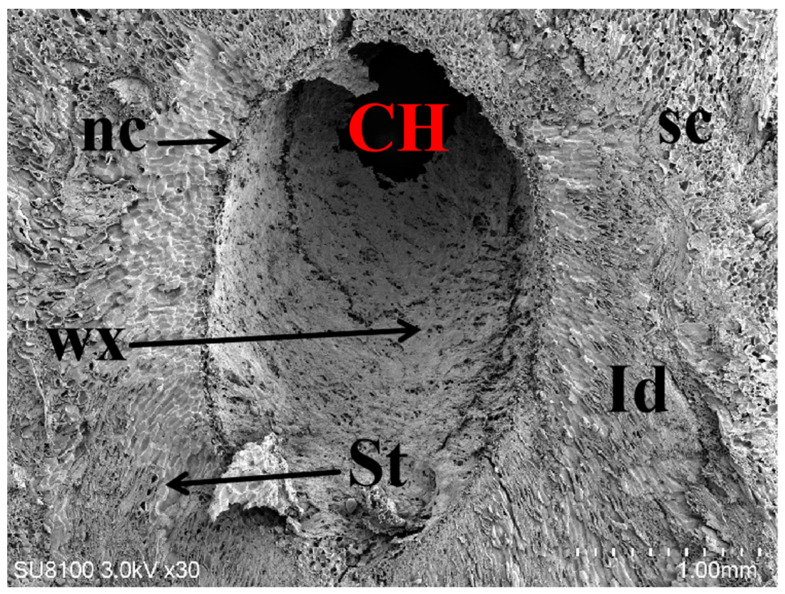
Scanning electron micrograph of the internal structure of a stage E chestnut gall induced by GWDK. nc: Nutrient layer, CH: Gall chamber, wx: Wax, St: Soft tissue with vacuoles, sc: Sclerenchyma epidermal layer of galls, Id: Internal dense tissue. Scale bars = 1.00 mm.

As the gall chambers progressed, significant alterations were observed in the cells adjacent to the chamber throughout various stages. During stage A, the cells neighboring the chamber underwent rapid shrinkage, whereas parenchyma cells exhibited extensive proliferation. Notably, some parenchyma cells exhibited a honeycomb-like appearance on their surface (Figure 9(A3)), leading to a reduction in cell spacing and increased cell density. Furthermore, the compression of inner cells resulted in the formation of the initial gall chamber, exhibiting rough, uneven, and irregular edges (Figure 9(A2)). During stage B, parenchyma cells surrounding the gall chamber exhibited a continued increase in density, accompanied by the emergence of numerous tiny pores on their surfaces (Figure 9(B3)). It is suggested that the innermost parenchyma cells initiated disassembly, resulting in thinner cells surrounding the main gall chamber and the appearance of small cavities (Figure 9(B2)). Widespread disintegration of parenchyma cells occurred, concurrent with the emergence of cotton-tufted objects within cells. These cotton-tufted objects subsequently developed into nutrient layer cells, and the merging of various-sized cavities resulted in the formation of the main gall chamber with smooth outer edges (Figure 9(C2,C3)). Parenchyma cells underwent breakdown and disintegration, with fusion and lignification observed in some cells. During the pupal stage, nutrient absorption from host plants ceased within the gall chambers, resulting in the accumulation of significant flocculent material (the product of larval emergence) within these chambers. Nutritive cells located on the exterior of the galls transformed into phloem and cambium cells, resulting in the thickening and hardening of the galls’ outer walls. Prior to cessation, the gall chamber area expanded to its maximum capacity (Figure 9(D2, D3)). At stage E, the parenchyma cells underwent complete disintegration and collapse, ultimately fusing to create a membrane structure (Figure 9(E3)). The cells within the nutrient layer dried out and perished, resulting in a darkened coloration of deposits (Figure 10). Wax-like substances emerged on the inner walls of the gall chambers. Concurrently, the sachyte tissues differentiated into dense internal tissues, while soft tissues containing vacuoles appeared in the periphery, enclosed by a sclerenchyma epidermal layer (Figure 10).

## 3. Discussion

### 3.1. External Morphological Characteristics of Chestnut Galls and Development of GWDK

Gall growth is a dynamic process, and a gall’s internal structural characteristics change during the continuous growth stages, which also influences the laying of eggs and feeding behaviors of phytophagous insects [19]. The results of this experiment reveal that the GWDK exhibited a preference for laying eggs in undifferentiated meristems, such as shoots, young leaves, or buds. These meristem cells are characterized by their youth, rapid division rate, loose arrangement, and high nutrient content, all of which are conducive to gall formation and growth. Similar observations have been reported by Ding et al. [20], Wang [21], and Guo et al. [22]. Galls were primarily found in the upper regions of branches, although not densely concentrated, which may be attributed to the ease of access to plant photosynthetic products by gall-causing insects [23]. Based on the degree of differentiation in tissue structure, galls can be categorized into tissue and organ galls, with tissue gall formation being characterized by a high degree of tissue and structural differentiation [24].

### 3.2. Microscopic Observation of Internal Gall Structure

The internal structure of galls is highly differentiated, exhibiting significant differences compared to normal tissue. Galls primarily comprise an epidermis, protective layer, parenchyma layer, and gall chamber. The variation in the internal structure of galls is predominantly attributed to the supply of nutrients, while differences in the external structure are influenced by defense requirements [25,26,27]. Typically, the outer perimeter of insect galls is divided into distinct layers consisting of sclerenchyma layer, internal parenchyma tissue, vacuolar parenchyma, internal soft tissue, and nutrient layer [3,6,7].

During various different developmental stages, the internal structure of galls undergoes changes to cater to the larval growth and development requirements. In stage A of gall development, the epidermal and protective layers significantly thickened, resulting in the formation of the sclerenchyma layer, primarily composed of epidermal and sclerenchyma cells, offering protection to the developing GWDK larvae. The parenchyma was mainly composed of endogenous parenchyma cells. During stage B and stage C, the nuclei around the gall chambers enlarged, leading to the formation of outer and inner parenchyma cells. The extent of parenchyma cell proliferation determines the size and weight of galls. JARA, 2021 et al. [5] achieved consistent findings in their gall research. SEM observations reveal that the outer epidermis of galls exhibits a smooth and relaxed appearance during the initial stage, but elongates and may crack upon maturity to accommodate an expanded surface area. This phenomenon is commonly observed in other plant galls [28,29]. Larvae alter the cell expansion pattern of the gall epidermis, resulting in slender margins devoid of papillae. This is attributed to the hypertrophy and proliferation of procambial cells, which contribute to the increase in gall volume. Complex changes occur in the epidermis, reflecting the intricate nature of galls, which include variations in density, size, and morphology of the trichomes and stomata, among other epidermal characteristics [30,31]. Minor changes occur in the epidermis, whereas substantial alterations are observed in underlying tissues. These alterations include the emergence of new tissues, such as the sclerenchyma layer, as well as the rearrangement of nutrient cells and the vascular system. The procambium of galls also undergoes changes, involving the disintegration of parenchyma cells to form nutrient cells. Subsequently, these nutrient cells differentiate into multiple layers of cells with thick secondary walls, collectively referred to as the sclerenchyma, which provides structural rigidity [30]. In stages B and C, cells proximal to the inner layer of the GWDK galls exhibit dense cytoplasm and large nuclei. This observation suggests the presence of metabolically active nutritive cells, which disintegrate the inner layer of parenchyma cells, aligning with previous research findings [32]. The natural enemies of the GWDK larvae prefer to pierce the outer wall of the initiation gall and deposit their eggs within. Cooper and Rieske [33] found a negative correlation between the amount of sclerenchyma in each GWDK gall and the number of parasitic wasps. As the protective layer of the sclerenchyma decreases, the likelihood of a successful attack by an external parasite increases. This protective effect may stem from the rigid sclerenchyma cell walls, effectively shielding the GWDK larvae in galls from the oviposition of a natural enemy. In stages B and C, the cells proximal to the inner layer of the GWDK galls had dense metabolic cytoplasm and large nuclei. They functioned as metabolically active nutritive cells, responsible for disintegrating the inner layer of parenchyma cells. This observation concurs with the results of Ferreira et al. [30,34].

In ROS-stained sections, ROS accumulation was observed at various stages of gall development. Following larval invasion, a significant surge in ROS levels was observed at stage D, aligning with previous findings reported by Lin et al. [35] and Zhang et al. [36] in plants infected with pathogens and experiencing allergic reactions. Additionally, at stage C, a substantial accumulation of ROS was evident, exhibiting distinct regionalization patterns, despite the most rapid growth in gall dimension and weight occurring during this stage. The observed discrepancy between ROS accumulation and gall structural changes suggests that a threshold level of ROS accumulation is required to trigger significant alterations, resulting in a delayed nutritional outbreak until stage D. Oxidation outbreaks, similar to those observed during gall development, can arise from fungal, bacterial, and viral infections in plants, leading to severe damage to proteins, membrane lipids, DNA, and other cellular components [37]. Under normal physiological conditions, mitochondria and chloroplasts primarily contribute to ROS production. However, during pathogen-induced oxidation outbreaks, elevated ROS levels can also be detected outside cell membranes [38].

### 3.3. Effects of Gall on Host Organ Structure

The formation of galls results in the alteration of the original vascular system, leading to the emergence of several lateral vascular bundles or networks. This rearrangement may be influenced by fluctuations in gall growth rates throughout the development stages [30,39]. The branches of the vascular networks may relocate nutrients from the developing leaves to the gall through a compensatory hydraulic mechanism. The hydraulic compensation mechanism results in the accumulation of water and nutrients in galls, thus promoting gall growth by accumulating water within them. The changes in the vascular bundles affect the normal formation of the distal tissue of leaves and therefore may lead to changes in the strength of the source–sink relation, affecting the growth continuity of the branches and fruit production and thus reducing chestnut tree productivity. The variation in gall water content may explain the initial enlargement and subsequent shrinkage of the gall chamber [7]. Therefore, the subsequent decrease in tissue thickness of each layer may be caused either by the gradual disintegration of some nutritive and proliferating cells or by the reduction in cell volume due to a decrease in gall water content. This result is consistent with previous research performed by Hu et al. in a study on Asphondylia sp [40]. Galls contain parenchyma tissue cells and many easily proliferating vascular tissues, which directly affect the production and transport of host nutrients, making galls a new nutrient source–sink that can provide bidirectional nutrition for larval growth and development [41]. Swelling of the vacuole parenchyma and branches of vascular bundles may result in the transfer of water and light compounds to the galls rather than to the distal ends of branches and leaves. With continuous growth of the larvae and gall, there was gradual fading of the outer skin color and browning of the internal tissue. With consideration of the results of ROS staining, the changes were due to decreased activity of tissue antioxidant enzymes and weakened function of eliminating ROS and free radicals, and ROS accumulation can ultimately cause lipid peroxidation of cell membranes. Thus, the integrity of the cell membrane structure was damaged, cells were harmed, with effects on plant respiration and photosynthesis, and the aging and color degeneration of plant tissues were accelerated [35]. This study sheds light on the structural variations in GWDK-induced galls at different developmental stages, offering valuable insights into the formation process and underlying mechanisms. Further research integrating knowledge of the host plant and insect physiology and nutrition is essential to deepen our understanding of gall formation mechanisms and develop effective prevention and control strategies against GWDK infestations.

## 4. Materials and Methods

### 4.1. Materials

Galls induced by GWDK were harvested from 8-year-old Chinese chestnut (Castanea mollissima Blume) plants known as ‘Hongyouli (HL)’ in the Chinese chestnut germplasm resources bank, located in Nanjing, Jiangsu Province, China (31°14′6″ N, 118°22′12″ E) [42]. ‘HL’ exhibits susceptibility to GWDK infestation, resulting in the formation of galls on both leaves and buds. The bursting of chestnut leaf buds commenced on 15 March 2023. From this date onward, a total of 30 chestnut galls were collected from 6 Chinese chestnut trees (in same ages) at each developmental stage, and samples were taken at 7-day intervals. Fifteen galls were selected randomly from the 30 samples and used for internal structure observation [43]. Consequently, galls were obtained at five distinct stages: the initial stage (stage A), growth stage (stage B), differentiation stage (stage C), maturity stage (stage D), and lignification stage (stage E). Fresh samples were promptly frozen in liquid nitrogen upon collection and subsequently subjected to laboratory analysis. For optical microscopy, the samples were cleansed with distilled water and directly examined under a microscope. Samples designated for paraffin sectioning were preserved in FAA fixative solution (formaldehyde–acetic acid–50% ethanol = 1:1:18). Meanwhile, samples used for scanning electron microscopy (SEM) were preserved in glutaraldehyde.

### 4.2. Methods

#### 4.2.1. Observations of Gall Morphology

Gall features and overall appearance were captured using a Canon EOS R8 camera (Canon, Oita Prefecture, Japan), positioned directly on the tree trunk. Gall structural characteristics at various stages were observed and measured using a Moticam 2506 anatomical microscope (Olympus, Tokyo, Japan) in conjunction with the Olympus Motic Images Advanced 3.2 microscopic system (Olympus, Tokyo, Japan).

#### 4.2.2. Observations of Gall Structure

The Safranin O/Fast Green staining method described by Alvarez et al. [44] was employed. Safranin O solution was used to visualize lignified, thrombolyzed, and keratinized parts and chromatin in the nucleus, appearing red, while Fast Green stain highlighted cellulose cell tissue, appearing blue. This staining allowed for visualization of the distribution and changes in the outer peritissue cells of the gall chambers. Samples underwent dehydration in an increasing ethanol series, with isoamyl acetate used as an intermediate liquid medium for embedding in Paraplast^®^ (Leica, Weztlar, Germany). The paraffin blocks were then sliced into 12 µm thick sections using a rotary microtome, and these sections were affixed to slides. Following dewaxing with xylene, the sections were stained with Safranin O/Fast Green stain, dehydrated, and permanently mounted on microscope slides using Entellan^®^ (Leica, Weztlar, Germany).

Periodic acid–Schiff staining followed the method of Pan et al. [45]. Schiff reagent was used to highlight sugars and other substances in the tissue, appearing purplish red, while Hematoxylin stain solution illuminated the cytoplasm, appearing light red to red. This staining facilitated intuitive visualization of the distribution and flow of nutrients in the gall sections. The staining process involved routine dewaxing of slices to water, oxidation with Periodic acid solution for 10 min, rinsing with distilled water, staining with 35% Schiff reagent in the dark at room temperature for 15–20 min, rinsing with water, staining nuclei with 10% Hematoxylin stain solution for 2–5 min, rinsing with water, and mounting samples on microscope slides using conventional dehydrating transparency and neutral balsam seal. The slides were examined with a Nikon composite microscope (E600, Nikon, Tokyo, Japan) under bright field, converging fluorescence, and polarized light, and images were captured with a coupled digital camera.

#### 4.2.3. Staining for Reactive Oxygen Species (ROS) Accumulation in Frozen Sections

Reactive oxygen species (ROS) encompass a group of molecules crucial for cellular metabolism and signaling. While ROS aid in cellular defense mechanisms by clearing harmful oxidative stress, an imbalance between their production and elimination can lead to cell damage. ROS distribution in gall tissues can be visualized through ROS staining, as outlined below. (1) Self-fluorescence quenching of tissues: Frozen sections were brought to room temperature, and moisture was regulated. Circles were drawn around the tissue with a histochemical pen, and a self-fluorescence quencher was applied for 5 min, followed by rinsing with water for 10 min. (2) Dyeing: ROS dye solution (Sigma Aldrich Shanghai Trading Co Ltd, Shanghai, China.) was added to the ring and incubated at 37 °C in a dark incubator for 30 min. (3) DAPI restaining nuclei: The slide was placed in a 2 µg/mL PBS solution (pH 7.4, Servicebio, Wuhan, China) and washed by shaking on the decolorizing shaker for 3 times, 5 min each. DAPI dye solution (Servicebio, Wuhan, China) was added and incubated at room temperature away from light for 10 min. (4) Sealing: The slide was placed in PBS (pH 7.4) and washed by shaking on the decolorizing table for 3 times, 5 min each. Anti-fluorescence quenching sealing tablets were applied. (5) Image acquisition: DAPI excitation wavelength ranged from 330 to 380 nm, with emission wavelength at 420 nm. The excitation wavelength for 488 (Green fluorescein) was 465–495 nm, and emission wavelength was 515–555 nm. For CY3 (Red fluorescein), excitation wavelength was 510–560 nm, with emission wavelength at 590 nm. CY5 (Pink fluorescein) excitation wavelength was 608–648 nm, and emission wavelength was 672–712 nm. DAPI staining rendered the nucleus blue, and red indicated a positive indication of ROS.

#### 4.2.4. Scanning Electron Microscope (SEM) Observations

According to the method of Borowiec et al. [46], galls were dissected along the gall body and then fixed with 3% glutaraldehyde at 4 °C for 12 h. Afterward, the samples underwent three rinses with PBS, each lasting 15 min. Next, the samples were fixed with 1% osmic acid at 4 °C for 1.5 h, followed by another round of rinsing with PBS three times, with each rinse lasting 15 min. The samples were then dehydrated using a series of ethanol concentrations, 30%, 50%, 70%, 80%, and 90%, with each concentration applied once for 15 min. Dehydration was completed with three rounds of 100% anhydrous ethanol, with each round lasting 15 min. Hexamethyldisilane (HM-DS) was replaced twice for 15 min each time. This step ensured rapid and thorough replacement of the dehydrating agent and residual water in the sample, facilitating quick and complete drying to prevent tissue damage from atmospheric pressure. Subsequently, the samples were vacuum-dried, coated with gold spray, and examined under a scanning electron microscope (SEM, Leica, Weztlar, Germany).

## 5. Conclusions

This study examined the developmental stages of galls induced by the GWDK in Chinese chestnut plants ‘Hongyouli (HL)’. The oviposition of the chestnut gall wasp prompted changes in host plant tissues, with gall formation predominantly occurring in young tissues such as leaves and buds. GWDK infestation induced the formation of gall structures comprising parenchyma, protective, and epidermal layers. The study also highlighted the role of reactive oxygen species (ROS) in gall development. Infestation by GWDK weakened the host’s ROS elimination ability, leading to ROS accumulation and subsequent peroxidation of the cell walls. This process compromised the host’s resistance, contributing to gall formation. Moreover, the parenchyma layer played a crucial role in nutrient supply, thickening noticeably during the differentiation stage. Changes in the thickness of the outer protective and epidermal layers were observed across different growth stages. The finding underscores the importance of integrating knowledge of host plant and insect physiology and nutrition to elucidate gall development mechanisms further. For instance, investigating antioxidant enzyme activities in GWDK larvae and host plants at different gall development stages could provide valuable insights for GWDK prevention and control strategies.

## Data Availability

The data that support the findings of this study are available from the corresponding author upon reasonable request.
